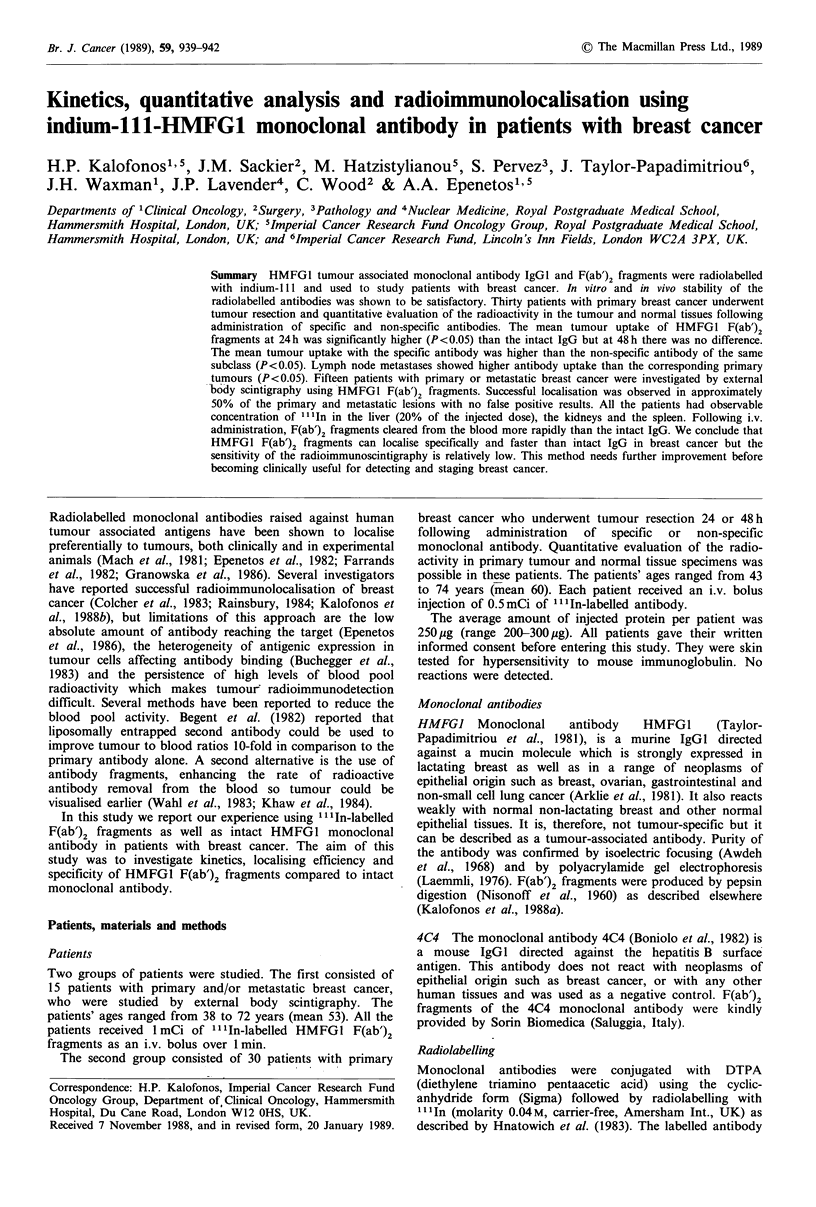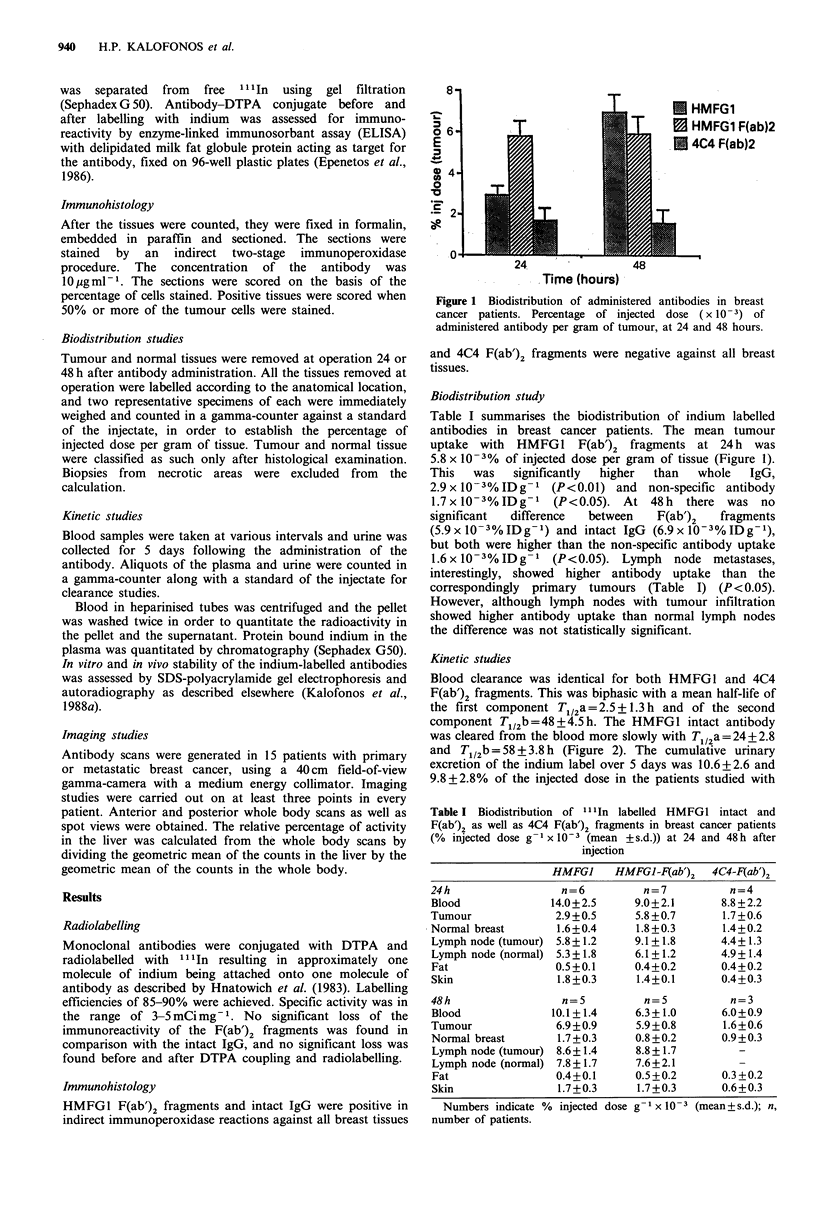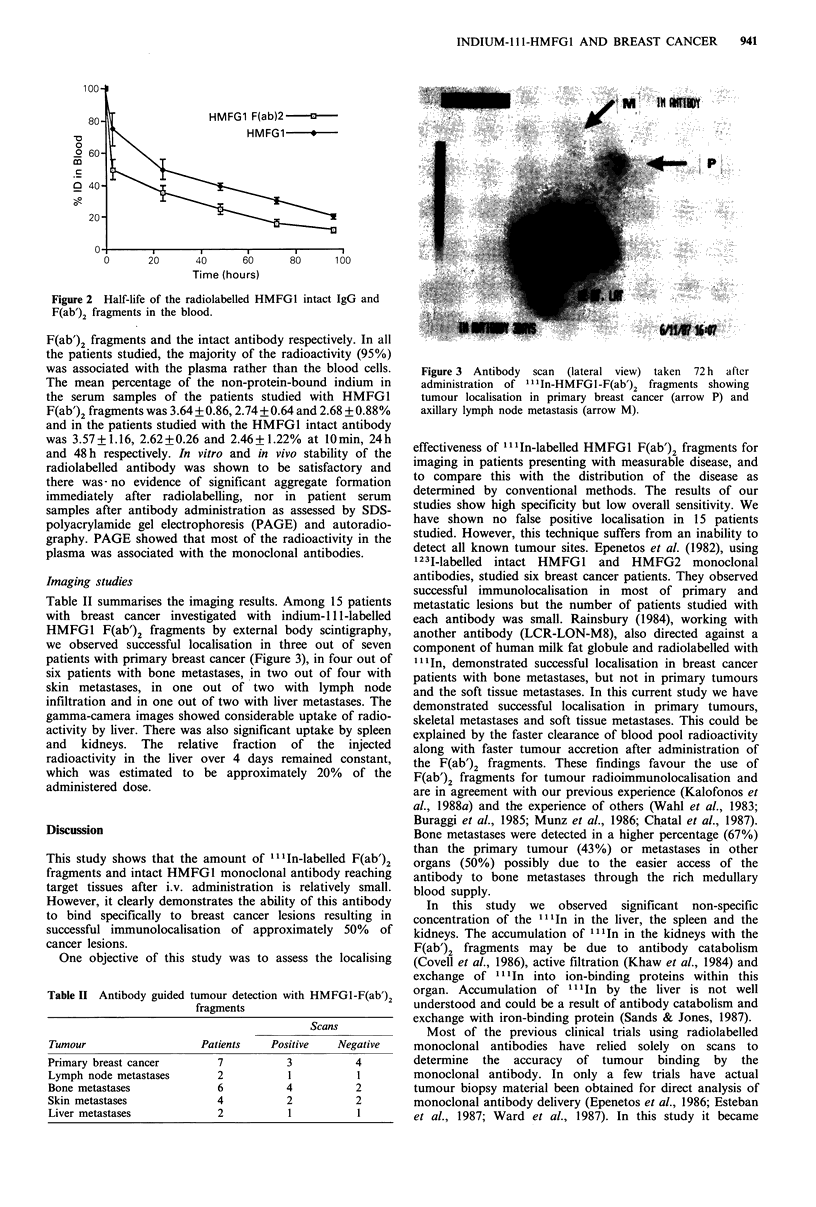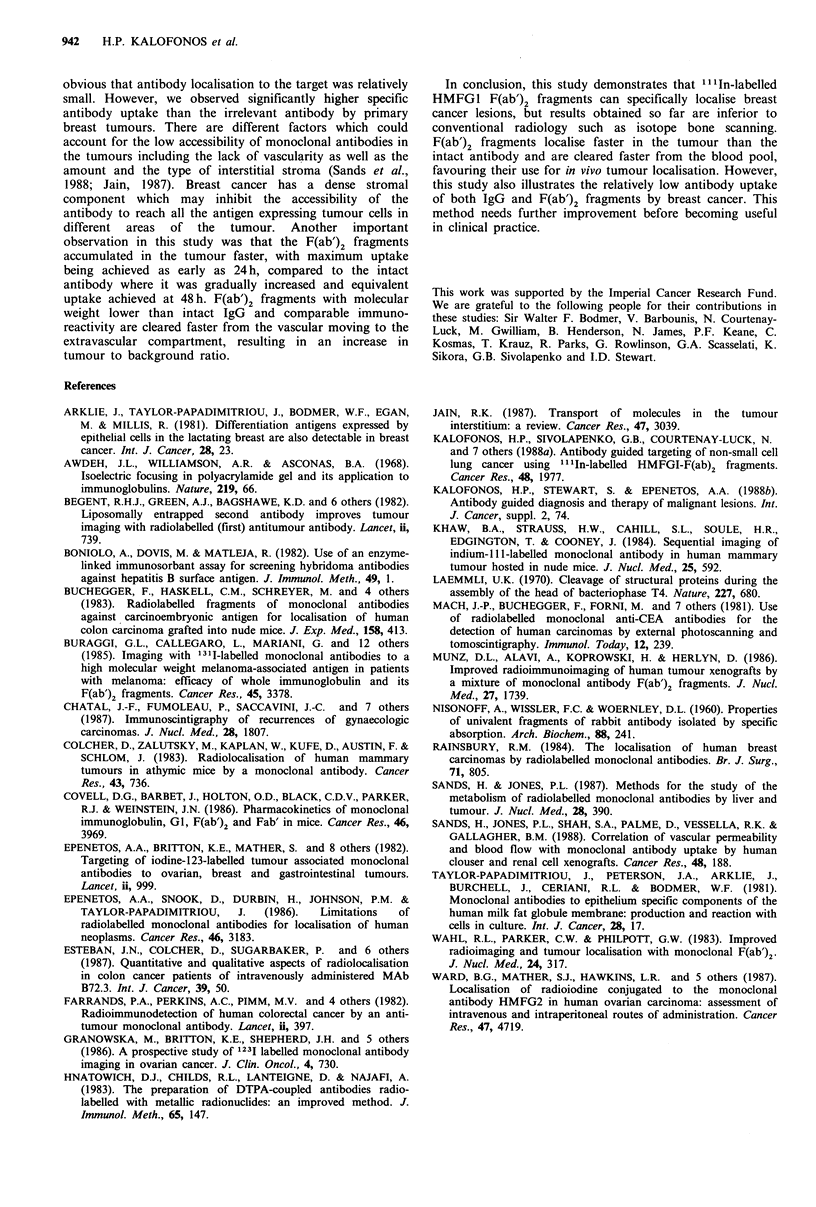# Kinetics, quantitative analysis and radioimmunolocalization using indium-111-HMFG1 monoclonal antibody in patients with breast cancer.

**DOI:** 10.1038/bjc.1989.199

**Published:** 1989-06

**Authors:** H. P. Kalofonos, J. M. Sackier, M. Hatzistylianou, S. Pervez, J. Taylor-Papadimitriou, J. H. Waxman, J. P. Lavender, C. Wood, A. A. Epenetos

**Affiliations:** Department of Clinical Oncology, Royal Postgraduate Medical School, Hammersmith Hospital, London, UK.

## Abstract

**Images:**


*Br. J. Cancer* (1989), **59**, 939–942 © The Macmillan Press Ltd., 1989

### Kinetics, quantitative analysis and radioimmunolocalisation using indium-111-HMFG1 monoclonal antibody in patients with breast cancer

H.P. Kalofonos[1,5], J.M. Sackier[2], M. Hatzistylianou[5], S. Pervez[3], J. Taylor-Papadimitriou[6], J.H. Waxman[1], J.P. Lavender[4], C. Wood[2] & A.A. Epenetos[1,5]

*Departments of [1]Clinical Oncology, [2]Surgery, [3]Pathology and [4]Nuclear Medicine, Royal Postgraduate Medical School, Hammersmith Hospital, London, UK; [5]Imperial Cancer Research Fund Oncology Group, Royal Postgraduate Medical School, Hammersmith Hospital, London, UK; and [6]Imperial Cancer Research Fund, Lincoln's Inn Fields, London WC2A 3PX, UK.*

**Summary** HMFG1 tumour associated monoclonal antibody IgG1 and $F(ab')_2$ fragments were radiolabelled with indium-111 and used to study patients with breast cancer. *In vitro* and *in vivo* stability of the radiolabelled antibodies was shown to be satisfactory. Thirty patients with primary breast cancer underwent tumour resection and quantitative evaluation of the radioactivity in the tumour and normal tissues following administration of specific and non-specific antibodies. The mean tumour uptake of HMFG1 $F(ab')_2$ fragments at 24 h was significantly higher ($P<0.05$) than the intact IgG but at 48 h there was no difference. The mean tumour uptake with the specific antibody was higher than the non-specific antibody of the same subclass ($P<0.05$). Lymph node metastases showed higher antibody uptake than the corresponding primary tumours ($P<0.05$). Fifteen patients with primary or metastatic breast cancer were investigated by external body scintigraphy using HMFG1 $F(ab')_2$ fragments. Successful localisation was observed in approximately 50% of the primary and metastatic lesions with no false positive results. All the patients had observable concentration of $^{111}$In in the liver (20% of the injected dose), the kidneys and the spleen. Following i.v. administration, $F(ab')_2$ fragments cleared from the blood more rapidly than the intact IgG. We conclude that HMFG1 $F(ab')_2$ fragments can localise specifically and faster than intact IgG in breast cancer but the sensitivity of the radioimmunoscintigraphy is relatively low. This method needs further improvement before becoming clinically useful for detecting and staging breast cancer.

Radiolabelled monoclonal antibodies raised against human tumour associated antigens have been shown to localise preferentially to tumours, both clinically and in experimental animals (Mach *et al.*, 1981; Epenetos *et al.*, 1982; Farrands *et al.*, 1982; Granowska *et al.*, 1986). Several investigators have reported successful radioimmunolocalisation of breast cancer (Colcher *et al.*, 1983; Rainsbury, 1984; Kalofonos *et al.*, 1988*b*), but limitations of this approach are the low absolute amount of antibody reaching the target (Epenetos *et al.*, 1986), the heterogeneity of antigenic expression in tumour cells affecting antibody binding (Buchegger *et al.*, 1983) and the persistence of high levels of blood pool radioactivity which makes tumour radioimmunodetection difficult. Several methods have been reported to reduce the blood pool activity. Begent *et al.* (1982) reported that liposomally entrapped second antibody could be used to improve tumour to blood ratios 10-fold in comparison to the primary antibody alone. A second alternative is the use of antibody fragments, enhancing the rate of radioactive antibody removal from the blood so tumour could be visualised earlier (Wahl *et al.*, 1983; Khaw *et al.*, 1984).

In this study we report our experience using $^{111}$In-labelled $F(ab')_2$ fragments as well as intact HMFG1 monoclonal antibody in patients with breast cancer. The aim of this study was to investigate kinetics, localising efficiency and specificity of HMFG1 $F(ab')_2$ fragments compared to intact monoclonal antibody.

### Patients, materials and methods

### *Patients*

Two groups of patients were studied. The first consisted of 15 patients with primary and/or metastatic breast cancer, who were studied by external body scintigraphy. The patients' ages ranged from 38 to 72 years (mean 53). All the patients received 1 mCi of $^{111}$In-labelled HMFG1 $F(ab')_2$ fragments as an i.v. bolus over 1 min.

The second group consisted of 30 patients with primary breast cancer who underwent tumour resection 24 or 48 h following administration of specific or non-specific monoclonal antibody. Quantitative evaluation of the radioactivity in primary tumour and normal tissue specimens was possible in these patients. The patients' ages ranged from 43 to 74 years (mean 60). Each patient received an i.v. bolus injection of 0.5 mCi of $^{111}$In-labelled antibody.

The average amount of injected protein per patient was 250 μg (range 200–300 μg). All patients gave their written informed consent before entering this study. They were skin tested for hypersensitivity to mouse immunoglobulin. No reactions were detected.

### *Monoclonal antibodies*

*HMFG1* Monoclonal antibody HMFG1 (Taylor-Papadimitriou *et al.*, 1981), is a murine IgG1 directed against a mucin molecule which is strongly expressed in lactating breast as well as in a range of neoplasms of epithelial origin such as breast, ovarian, gastrointestinal and non-small cell lung cancer (Arklie *et al.*, 1981). It also reacts weakly with normal non-lactating breast and other normal epithelial tissues. It is, therefore, not tumour-specific but it can be described as a tumour-associated antibody. Purity of the antibody was confirmed by isoelectric focusing (Awdeh *et al.*, 1968) and by polyacrylamide gel electrophoresis (Laemmli, 1976). $F(ab')_2$ fragments were produced by pepsin digestion (Nisonoff *et al.*, 1960) as described elsewhere (Kalofonos *et al.*, 1988*a*).

*4C4* The monoclonal antibody 4C4 (Boniolo *et al.*, 1982) is a mouse IgG1 directed against the hepatitis B surface antigen. This antibody does not react with neoplasms of epithelial origin such as breast cancer, or with any other human tissues and was used as a negative control. $F(ab')_2$ fragments of the 4C4 monoclonal antibody were kindly provided by Sorin Biomedica (Saluggia, Italy).

### *Radiolabelling*

Monoclonal antibodies were conjugated with DTPA (diethylene triamino pentaacetic acid) using the cyclic-anhydride form (Sigma) followed by radiolabelling with $^{111}$In (molarity 0.04 M, carrier-free, Amersham Int., UK) as described by Hnatowich *et al.* (1983). The labelled antibody

Correspondence: H.P. Kalofonos, Imperial Cancer Research Fund Oncology Group, Department of Clinical Oncology, Hammersmith Hospital, Du Cane Road, London W12 0HS, UK.
Received 7 November 1988, and in revised form, 20 January 1989.

was separated from free $^{111}In$ using gel filtration (Sephadex G 50). Antibody–DTPA conjugate before and after labelling with indium was assessed for immunoreactivity by enzyme-linked immunosorbant assay (ELISA) with delipidated milk fat globule protein acting as target for the antibody, fixed on 96-well plastic plates (Epenetos *et al.*, 1986).

*Immunohistology*

After the tissues were counted, they were fixed in formalin, embedded in paraffin and sectioned. The sections were stained by an indirect two-stage immunoperoxidase procedure. The concentration of the antibody was $10\,\mu g\,ml^{-1}$. The sections were scored on the basis of the percentage of cells stained. Positive tissues were scored when 50% or more of the tumour cells were stained.

*Biodistribution studies*

Tumour and normal tissues were removed at operation 24 or 48 h after antibody administration. All the tissues removed at operation were labelled according to the anatomical location, and two representative specimens of each were immediately weighed and counted in a gamma-counter against a standard of the injectate, in order to establish the percentage of injected dose per gram of tissue. Tumour and normal tissue were classified as such only after histological examination. Biopsies from necrotic areas were excluded from the calculation.

*Kinetic studies*

Blood samples were taken at various intervals and urine was collected for 5 days following the administration of the antibody. Aliquots of the plasma and urine were counted in a gamma-counter along with a standard of the injectate for clearance studies.

Blood in heparinised tubes was centrifuged and the pellet was washed twice in order to quantitate the radioactivity in the pellet and the supernatant. Protein bound indium in the plasma was quantitated by chromatography (Sephadex G50). *In vitro* and *in vivo* stability of the indium-labelled antibodies was assessed by SDS-polyacrylamide gel electrophoresis and autoradiography as described elsewhere (Kalofonos *et al.*, 1988*a*).

*Imaging studies*

Antibody scans were generated in 15 patients with primary or metastatic breast cancer, using a 40 cm field-of-view gamma-camera with a medium energy collimator. Imaging studies were carried out on at least three points in every patient. Anterior and posterior whole body scans as well as spot views were obtained. The relative percentage of activity in the liver was calculated from the whole body scans by dividing the geometric mean of the counts in the liver by the geometric mean of the counts in the whole body.

### Results

*Radiolabelling*

Monoclonal antibodies were conjugated with DTPA and radiolabelled with $^{111}In$ resulting in approximately one molecule of indium being attached onto one molecule of antibody as described by Hnatowich *et al.* (1983). Labelling efficiencies of 85–90% were achieved. Specific activity was in the range of $3\text{–}5\,mCi\,mg^{-1}$. No significant loss of the immunoreactivity of the $F(ab')_2$ fragments was found in comparison with the intact IgG, and no significant loss was found before and after DTPA coupling and radiolabelling.

*Immunohistology*

HMFG1 $F(ab')_2$ fragments and intact IgG were positive in indirect immunoperoxidase reactions against all breast tissues

**Figure 1** Biodistribution of administered antibodies in breast cancer patients. Percentage of injected dose ($\times 10^{-3}$) of administered antibody per gram of tumour, at 24 and 48 hours.

and 4C4 $F(ab')_2$ fragments were negative against all breast tissues.

*Biodistribution study*

Table I summarises the biodistribution of indium labelled antibodies in breast cancer patients. The mean tumour uptake with HMFG1 $F(ab')_2$ fragments at 24 h was $5.8 \times 10^{-3}$% of injected dose per gram of tissue (Figure 1). This was significantly higher than whole IgG, $2.9 \times 10^{-3}\%\,ID\,g^{-1}$ ($P<0.01$) and non-specific antibody $1.7 \times 10^{-3}\%\,ID\,g^{-1}$ ($P<0.05$). At 48 h there was no significant difference between $F(ab')_2$ fragments ($5.9 \times 10^{-3}\%\,ID\,g^{-1}$) and intact IgG ($6.9 \times 10^{-3}\%\,ID\,g^{-1}$), but both were higher than the non-specific antibody uptake $1.6 \times 10^{-3}\%\,ID\,g^{-1}$ ($P<0.05$). Lymph node metastases, interestingly, showed higher antibody uptake than the correspondingly primary tumours (Table I) ($P<0.05$). However, although lymph nodes with tumour infiltration showed higher antibody uptake than normal lymph nodes the difference was not statistically significant.

*Kinetic studies*

Blood clearance was identical for both HMFG1 and 4C4 $F(ab')_2$ fragments. This was biphasic with a mean half-life of the first component $T_{1/2}a = 2.5 \pm 1.3$ h and of the second component $T_{1/2}b = 48 \pm 4.5$ h. The HMFG1 intact antibody was cleared from the blood more slowly with $T_{1/2}a = 24 \pm 2.8$ and $T_{1/2}b = 58 \pm 3.8$ h (Figure 2). The cumulative urinary excretion of the indium label over 5 days was $10.6 \pm 2.6$ and $9.8 \pm 2.8$% of the injected dose in the patients studied with

**Table I** Biodistribution of $^{111}In$ labelled HMFG1 intact and $F(ab')_2$ as well as 4C4 $F(ab')_2$ fragments in breast cancer patients (% injected dose $g^{-1} \times 10^{-3}$ (mean ±s.d.)) at 24 and 48 h after injection

| | *HMFG1* | *HMFG1-F(ab')₂* | *4C4-F(ab')₂* |
|---|---|---|---|
| *24 h* | $n=6$ | $n=7$ | $n=4$ |
| Blood | 14.0±2.5 | 9.0±2.1 | 8.8±2.2 |
| Tumour | 2.9±0.5 | 5.8±0.7 | 1.7±0.6 |
| Normal breast | 1.6±0.4 | 1.8±0.3 | 1.4±0.2 |
| Lymph node (tumour) | 5.8±1.2 | 9.1±1.8 | 4.4±1.3 |
| Lymph node (normal) | 5.3±1.8 | 6.1±1.2 | 4.9±1.4 |
| Fat | 0.5±0.1 | 0.4±0.2 | 0.4±0.2 |
| Skin | 1.8±0.3 | 1.4±0.1 | 0.4±0.3 |
| *48 h* | $n=5$ | $n=5$ | $n=3$ |
| Blood | 10.1±1.4 | 6.3±1.0 | 6.0±0.9 |
| Tumour | 6.9±0.9 | 5.9±0.8 | 1.6±0.6 |
| Normal breast | 1.7±0.3 | 0.8±0.2 | 0.9±0.3 |
| Lymph node (tumour) | 8.6±1.4 | 8.8±1.7 | – |
| Lymph node (normal) | 7.8±1.7 | 7.6±2.1 | – |
| Fat | 0.4±0.1 | 0.5±0.2 | 0.3±0.2 |
| Skin | 1.7±0.3 | 1.7±0.3 | 0.6±0.3 |

Numbers indicate % injected dose $g^{-1} \times 10^{-3}$ (mean±s.d.); $n$, number of patients.

**Figure 2** Half-life of the radiolabelled HMFG1 intact IgG and $F(ab')_2$ fragments in the blood.

$F(ab')_2$ fragments and the intact antibody respectively. In all the patients studied, the majority of the radioactivity (95%) was associated with the plasma rather than the blood cells. The mean percentage of the non-protein-bound indium in the serum samples of the patients studied with HMFG1 $F(ab')_2$ fragments was $3.64 \pm 0.86$, $2.74 \pm 0.64$ and $2.68 \pm 0.88$% and in the patients studied with the HMFG1 intact antibody was $3.57 \pm 1.16$, $2.62 \pm 0.26$ and $2.46 \pm 1.22$% at 10 min, 24 h and 48 h respectively. *In vitro* and *in vivo* stability of the radiolabelled antibody was shown to be satisfactory and there was no evidence of significant aggregate formation immediately after radiolabelling, nor in patient serum samples after antibody administration as assessed by SDS-polyacrylamide gel electrophoresis (PAGE) and autoradiography. PAGE showed that most of the radioactivity in the plasma was associated with the monoclonal antibodies.

### *Imaging studies*

Table II summarises the imaging results. Among 15 patients with breast cancer investigated with indium-111-labelled HMFG1 $F(ab')_2$ fragments by external body scintigraphy, we observed successful localisation in three out of seven patients with primary breast cancer (Figure 3), in four out of six patients with bone metastases, in two out of four with skin metastases, in one out of two with lymph node infiltration and in one out of two with liver metastases. The gamma-camera images showed considerable uptake of radioactivity by liver. There was also significant uptake by spleen and kidneys. The relative fraction of the injected radioactivity in the liver over 4 days remained constant, which was estimated to be approximately 20% of the administered dose.

### Discussion

This study shows that the amount of $^{111}$In-labelled $F(ab')_2$ fragments and intact HMFG1 monoclonal antibody reaching target tissues after i.v. administration is relatively small. However, it clearly demonstrates the ability of this antibody to bind specifically to breast cancer lesions resulting in successful immunolocalisation of approximately 50% of cancer lesions.

One objective of this study was to assess the localising

**Table II** Antibody guided tumour detection with HMFG1-$F(ab')_2$ fragments

| | | Scans | |
|---|---|---|---|
| *Tumour* | *Patients* | *Positive* | *Negative* |
| Primary breast cancer | 7 | 3 | 4 |
| Lymph node metastases | 2 | 1 | 1 |
| Bone metastases | 6 | 4 | 2 |
| Skin metastases | 4 | 2 | 2 |
| Liver metastases | 2 | 1 | 1 |

**Figure 3** Antibody scan (lateral view) taken 72 h after administration of $^{111}$In-HMFG1-$F(ab')_2$ fragments showing tumour localisation in primary breast cancer (arrow P) and axillary lymph node metastasis (arrow M).

effectiveness of $^{111}$In-labelled HMFG1 $F(ab')_2$ fragments for imaging in patients presenting with measurable disease, and to compare this with the distribution of the disease as determined by conventional methods. The results of our studies show high specificity but low overall sensitivity. We have shown no false positive localisation in 15 patients studied. However, this technique suffers from an inability to detect all known tumour sites. Epenetos *et al.* (1982), using $^{123}$I-labelled intact HMFG1 and HMFG2 monoclonal antibodies, studied six breast cancer patients. They observed successful immunolocalisation in most of primary and metastatic lesions but the number of patients studied with each antibody was small. Rainsbury (1984), working with another antibody (LCR-LON-M8), also directed against a component of human milk fat globule and radiolabelled with $^{111}$In, demonstrated successful localisation in breast cancer patients with bone metastases, but not in primary tumours and the soft tissue metastases. In this current study we have demonstrated successful localisation in primary tumours, skeletal metastases and soft tissue metastases. This could be explained by the faster clearance of blood pool radioactivity along with faster tumour accretion after administration of the $F(ab')_2$ fragments. These findings favour the use of $F(ab')_2$ fragments for tumour radioimmunolocalisation and are in agreement with our previous experience (Kalofonos *et al.*, 1988*a*) and the experience of others (Wahl *et al.*, 1983; Buraggi *et al.*, 1985; Munz *et al.*, 1986; Chatal *et al.*, 1987). Bone metastases were detected in a higher percentage (67%) than the primary tumour (43%) or metastases in other organs (50%) possibly due to the easier access of the antibody to bone metastases through the rich medullary blood supply.

In this study we observed significant non-specific concentration of the $^{111}$In in the liver, the spleen and the kidneys. The accumulation of $^{111}$In in the kidneys with the $F(ab')_2$ fragments may be due to antibody catabolism (Covell *et al.*, 1986), active filtration (Khaw *et al.*, 1984) and exchange of $^{111}$In into ion-binding proteins within this organ. Accumulation of $^{111}$In by the liver is not well understood and could be a result of antibody catabolism and exchange with iron-binding protein (Sands & Jones, 1987).

Most of the previous clinical trials using radiolabelled monoclonal antibodies have relied solely on scans to determine the accuracy of tumour binding by the monoclonal antibody. In only a few trials have actual tumour biopsy material been obtained for direct analysis of monoclonal antibody delivery (Epenetos *et al.*, 1986; Esteban *et al.*, 1987; Ward *et al.*, 1987). In this study it became obvious that antibody localisation to the target was relatively small. However, we observed significantly higher specific antibody uptake than the irrelevant antibody by primary breast tumours. There are different factors which could account for the low accessibility of monoclonal antibodies in the tumours including the lack of vascularity as well as the amount and the type of interstitial stroma (Sands *et al.*, 1988; Jain, 1987). Breast cancer has a dense stromal component which may inhibit the accessibility of the antibody to reach all the antigen expressing tumour cells in different areas of the tumour. Another important observation in this study was that the $F(ab')_2$ fragments accumulated in the tumour faster, with maximum uptake being achieved as early as 24 h, compared to the intact antibody where it was gradually increased and equivalent uptake achieved at 48 h. $F(ab')_2$ fragments with molecular weight lower than intact IgG and comparable immunoreactivity are cleared faster from the vascular moving to the extravascular compartment, resulting in an increase in tumour to background ratio.

In conclusion, this study demonstrates that $^{111}$In-labelled HMFG1 $F(ab')_2$ fragments can specifically localise breast cancer lesions, but results obtained so far are inferior to conventional radiology such as isotope bone scanning. $F(ab')_2$ fragments localise faster in the tumour than the intact antibody and are cleared faster from the blood pool, favouring their use for *in vivo* tumour localisation. However, this study also illustrates the relatively low antibody uptake of both IgG and $F(ab')_2$ fragments by breast cancer. This method needs further improvement before becoming useful in clinical practice.

This work was supported by the Imperial Cancer Research Fund. We are grateful to the following people for their contributions in these studies: Sir Walter F. Bodmer, V. Barbounis, N. Courtenay-Luck, M. Gwilliam, B. Henderson, N. James, P.F. Keane, C. Kosmas, T. Krauz, R. Parks, G. Rowlinson, G.A. Scasselati, K. Sikora, G.B. Sivolapenko and I.D. Stewart.

### References

ARKLIE, J., TAYLOR-PAPADIMITRIOU, J., BODMER, W.F., EGAN, M. & MILLIS, R. (1981). Differentiation antigens expressed by epithelial cells in the lactating breast are also detectable in breast cancer. *Int. J. Cancer*, **28**, 23.

AWDEH, J.L., WILLIAMSON, A.R. & ASCONAS, B.A. (1968). Isoelectric focusing in polyacrylamide gel and its application to immunoglobulins. *Nature*, **219**, 66.

BEGENT, R.H.J., GREEN, A.J., BAGSHAWE, K.D. and 6 others (1982). Liposomally entrapped second antibody improves tumour imaging with radiolabelled (first) antitumour antibody. *Lancet*, **ii**, 739.

BONIOLO, A., DOVIS, M. & MATLEJA, R. (1982). Use of an enzyme-linked immunosorbant assay for screening hybridoma antibodies against hepatitis B surface antigen. *J. Immunol. Meth.*, **49**, 1.

BUCHEGGER, F., HASKELL, C.M., SCHREYER, M. and 4 others (1983). Radiolabelled fragments of monoclonal antibodies against carcinoembryonic antigen for localisation of human colon carcinoma grafted into nude mice. *J. Exp. Med.*, **158**, 413.

BURAGGI, G.L., CALLEGARO, L., MARIANI, G. and 12 others (1985). Imaging with $^{131}$I-labelled monoclonal antibodies to a high molecular weight melanoma-associated antigen in patients with melanoma: efficacy of whole immunoglobulin and its $F(ab')_2$ fragments. *Cancer Res.*, **45**, 3378.

CHATAL, J.-F., FUMOLEAU, P., SACCAVINI, J.-C. and 7 others (1987). Immunoscintigraphy of recurrences of gynaecologic carcinomas. *J. Nucl. Med.*, **28**, 1807.

COLCHER, D., ZALUTSKY, M., KAPLAN, W., KUFE, D., AUSTIN, F. & SCHLOM, J. (1983). Radiolocalisation of human mammary tumours in athymic mice by a monoclonal antibody. *Cancer Res.*, **43**, 736.

COVELL, D.G., BARBET, J., HOLTON, O.D., BLACK, C.D.V., PARKER, R.J. & WEINSTEIN, J.N. (1986). Pharmacokinetics of monoclonal immunoglobulin, G1, $F(ab')_2$ and Fab' in mice. *Cancer Res.*, **46**, 3969.

EPENETOS, A.A., BRITTON, K.E., MATHER, S. and 8 others (1982). Targeting of iodine-123-labelled tumour associated monoclonal antibodies to ovarian, breast and gastrointestinal tumours. *Lancet*, **ii**, 999.

EPENETOS, A.A., SNOOK, D., DURBIN, H., JOHNSON, P.M. & TAYLOR-PAPADIMITRIOU, J. (1986). Limitations of radiolabelled monoclonal antibodies for localisation of human neoplasms. *Cancer Res.*, **46**, 3183.

ESTEBAN, J.N., COLCHER, D., SUGARBAKER, P. and 6 others (1987). Quantitative and qualitative aspects of radiolocalisation in colon cancer patients of intravenously administered MAb B72.3. *Int. J. Cancer*, **39**, 50.

FARRANDS, P.A., PERKINS, A.C., PIMM, M.V. and 4 others (1982). Radioimmunodetection of human colorectal cancer by an anti-tumour monoclonal antibody. *Lancet*, **ii**, 397.

GRANOWSKA, M., BRITTON, K.E., SHEPHERD, J.H. and 5 others (1986). A prospective study of $^{123}$I labelled monoclonal antibody imaging in ovarian cancer. *J. Clin. Oncol.*, **4**, 730.

HNATOWICH, D.J., CHILDS, R.L., LANTEIGNE, D. & NAJAFI, A. (1983). The preparation of DTPA-coupled antibodies radiolabelled with metallic radionuclides: an improved method. *J. Immunol. Meth.*, **65**, 147.

JAIN, R.K. (1987). Transport of molecules in the tumour interstitium: a review. *Cancer Res.*, **47**, 3039.

KALOFONOS, H.P., SIVOLAPENKO, G.B., COURTENAY-LUCK, N. and 7 others (1988*a*). Antibody guided targeting of non-small cell lung cancer using $^{111}$In-labelled HMFGI-F(ab)$_2$ fragments. *Cancer Res.*, **48**, 1977.

KALOFONOS, H.P., STEWART, S. & EPENETOS, A.A. (1988*b*). Antibody guided diagnosis and therapy of malignant lesions. *Int. J. Cancer*, suppl. 2, 74.

KHAW, B.A., STRAUSS, H.W., CAHILL, S.L., SOULE, H.R., EDGINGTON, T. & COONEY, J. (1984). Sequential imaging of indium-111-labelled monoclonal antibody in human mammary tumour hosted in nude mice. *J. Nucl. Med.*, **25**, 592.

LAEMMLI, U.K. (1970). Cleavage of structural proteins during the assembly of the head of bacteriophase T4. *Nature*, **227**, 680.

MACH, J.-P., BUCHEGGER, F., FORNI, M. and 7 others (1981). Use of radiolabelled monoclonal anti-CEA antibodies for the detection of human carcinomas by external photoscanning and tomoscintigraphy. *Immunol. Today*, **12**, 239.

MUNZ, D.L., ALAVI, A., KOPROWSKI, H. & HERLYN, D. (1986). Improved radioimmunoimaging of human tumour xenografts by a mixture of monoclonal antibody $F(ab')_2$ fragments. *J. Nucl. Med.*, **27**, 1739.

NISONOFF, A., WISSLER, F.C. & WOERNLEY, D.L. (1960). Properties of univalent fragments of rabbit antibody isolated by specific absorption. *Arch. Biochem.*, **88**, 241.

RAINSBURY, R.M. (1984). The localisation of human breast carcinomas by radiolabelled monoclonal antibodies. *Br. J. Surg.*, **71**, 805.

SANDS, H. & JONES, P.L. (1987). Methods for the study of the metabolism of radiolabelled monoclonal antibodies by liver and tumour. *J. Nucl. Med.*, **28**, 390.

SANDS, H., JONES, P.L., SHAH, S.A., PALME, D., VESSELLA, R.K. & GALLAGHER, B.M. (1988). Correlation of vascular permeability and blood flow with monoclonal antibody uptake by human clouser and renal cell xenografts. *Cancer Res.*, **48**, 188.

TAYLOR-PAPADIMITRIOU, J., PETERSON, J.A., ARKLIE, J., BURCHELL, J., CERIANI, R.L. & BODMER, W.F. (1981). Monoclonal antibodies to epithelium specific components of the human milk fat globule membrane: production and reaction with cells in culture. *Int. J. Cancer*, **28**, 17.

WAHL, R.L., PARKER, C.W. & PHILPOTT, G.W. (1983). Improved radioimaging and tumour localisation with monoclonal $F(ab')_2$. *J. Nucl. Med.*, **24**, 317.

WARD, B.G., MATHER, S.J., HAWKINS, L.R. and 5 others (1987). Localisation of radioiodine conjugated to the monoclonal antibody HMFG2 in human ovarian carcinoma: assessment of intravenous and intraperitoneal routes of administration. *Cancer Res.*, **47**, 4719.